# A Novel miRNA in Rice Associated with the Low Seed Setting Rate Symptom of Rice Stripe Virus

**DOI:** 10.3390/ijms24043675

**Published:** 2023-02-12

**Authors:** Quan Yuan, Yushan Zhai, Liya Zhou, Xuhong Ai, Jianping Chen, Fei Yan

**Affiliations:** 1 College of Plant Protection, Northwest Agriculture and Forestry University, Xianyang 712100, China; 2 State Key Laboratory for Managing Biotic and Chemical Threats to the Quality and Safety of Agro-Products, Key Laboratory of Biotechnology in Plant Protection of MARA and Zhejiang Province, Institute of Plant Virology, Ningbo University, Ningbo 315211, China

**Keywords:** rice stripe virus, miRNA, seed setting rate, symptoms, target

## Abstract

MicroRNAs play key regulatory roles in plant development. The changed pattern of miRNA expression is involved in the production of viral symptoms. Here, we showed that a small RNA, Seq119, a putative novel microRNA, is associated with the low seed setting rate, a viral symptom of rice stripe virus (RSV)-infected rice. The expression of Seq 119 was downregulated in RSV-infected rice. The overexpression of Seq119 in transgenic rice plants did not cause any obvious phenotypic changes in plant development. When the expression of Seq119 was suppressed in rice plants either by expressing a mimic target or by CRISPR/Cas editing, seed setting rates were extremely low, similar to the effects of RSV infection. The putative targets of Seq119 were then predicted. The overexpression of the target of Seq119 in rice caused a low seed setting rate, similar to that in Seq119-suppressed or edited rice plants. Consistently, the expression of the target was upregulated in Seq119-suppressed and edited rice plants. These results suggest that downregulated Seq119 is associated with the low seed setting rate symptom of the RSV in rice.

## 1. Introduction

MicroRNAs (miRNAs) play essential roles during the development of eukaryotes by targeting complementary mRNAs for degradation or translational repression [1,2]. In plants, miRNAs regulate developmental processes, including leaf morphogenesis and the development of roots and flowers [3,4,5,6,7,8]. Viral infection affects the pattern of miRNA expression in plants [9,10,11,12,13,14]. The changed pattern of miRNA expression is related to the production of viral symptoms [15,16,17]. For example, infection by tobamoviruses, potyviruses or potexviruses alters the accumulation of particular miRNAs in *Nicotiana tabacum* and symptom severity, appearing to be related to alterations in the levels of miR156, 160, 164, 166, 169 and 171 [15].

Seed setting is crucial for the yield and quality of rice grain. Rice miRNAs participate in seed setting. Wang et al. sequenced and analyzed the expression and evolution of rice miRNA genes participating in the pollen–pistil interaction that is an essential reproductive process for all flowering plants and identified a group of miRNAs participating in the interaction [18]. Osa-miR162a and osa-miR1873 have been reported to fine-tune rice immunity against *Magnaporthe oryzae* and affect yield traits [19,20]. The overexpression of osa-miR162a enhances rice resistance to *M. oryzae* accompanying the enhanced induction of defense-related genes. In contrast, blocking osa-miR162 by overexpressing a target mimic enhances susceptibility to the blast fungus associated with the compromised induction of defense-related gene expression. Moreover, transgenic lines overexpressing osa-miR162a have a decreased seed setting rate, resulting in slightly reduced yield per plant, whereas transgenic lines blocking osa-miR162 have an increased number of grains per panicle [19]. Osa-miR156 may also function in rice seed setting. A rice mutant with the elevated expression of osa-miR156e increases tiller number, reduces plant height, prolongs the heading date, results in low seed setting and leads to a small panicle size. Interestingly, the extent of impaired morphology is correlated with the expression level of osa-miR156e, which suggests that osa-miR156 could serve as a potential tool for modifying rice plant architecture [21].

Rice stripe disease is an economically important disease of rice caused by the rice stripe virus (RSV), which is transmitted by the small brown planthopper (SBPH) [22]. Infected rice plants have yellow stripes on their leaves, considerable stunting of the plants and a low seed setting rate, which causes a significant yield loss. In our previous report, we analyzed the expression pattern of miRNAs in RSV-infected rice and identified some novel miRNAs whose precursor sequences had not previously been described [23]. Moreover, we found that the downregulation of osa-miR171b is associated with the yellowing symptoms of the RSV [24]. However, there is little information on if and how other miRNAs are involved in RSV symptoms. Here, we identified that a small RNA Seq119, a putative novel microRNA, was associated with the low seed setting rate symptom of the RSV. The expression of Seq119 was downregulated in RSV-infected rice. The overexpression of Seq119 in transgenic rice plants did not cause any obvious change in developmental phenotype. However, the suppression of Seq119 by expressing a mimic target or by the CRISPR/Cas editing of plants resulted in extremely low seed setting rates similar to those in plants infected by the RSV. The putative targets of Seq119 were also predicted, and the overexpression of this target also caused a low seed setting rate. The results suggest that the downregulation of Seq119 is associated with the low seed setting rate symptom of the RSV in rice.

## 2. Results

### 2.1. Identification of Seq119 in Rice

**Figure 1:**
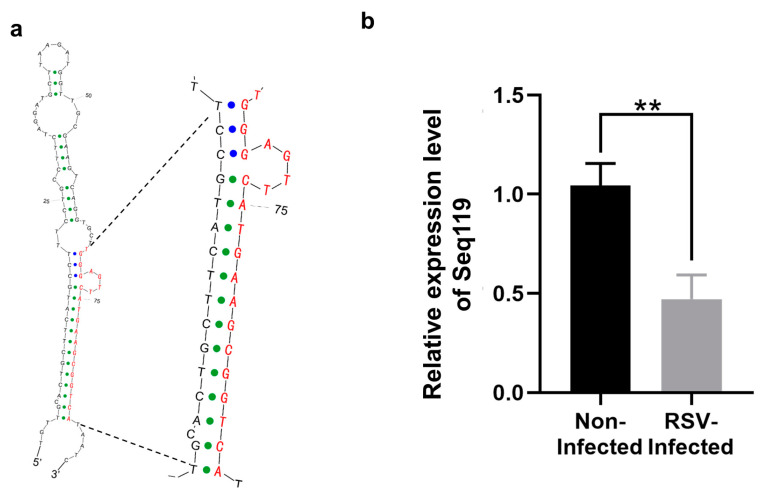
Predicted secondary structures of Seq119 precursor (**a**) and its downregulated expression in RSV-infected rice plants (**b**). Sequences of the putative mature miRNA in precursors are colored with red letters. The relative expression level of Seq119 in RSV-infected rice leaves was analyzed via qRT-PCR. Values are means ± SD. Student’s *t* test was used to test the significance of the difference (**, *p* < 0.01).

RSV-infected rice was shorter than non-infected rice and also had delayed heading in the field (Supplementary Figure S1a,b). Infected plants did not fully develop, resulting in an extremely reduced seed setting rate (Supplementary Figure S1c,d). In our previous report, we sequenced small RNAs in rice infected with the RSV for one week via Solexa sequencing. In libraries from both RSV-infected and virus-free plants, potential novel miRNAs were identified [23]. One small sequence identified, Seq119, was 21 nt in length (5′-UGGGAGUUCAUGAAGCGGUCA-3′), and its predicted precursor had a stable secondary structure (Figure 1a). This sequence could also be identified in six other reported libraries [25]. Moreover, its precursor sequence did not match the deposited rice miRNAs or miRNA precursors, which indicates that Seq119 might be a novel miRNA in rice. It had only one intergenic hit locus in the rice genome [23]. In our previous work, results from qRT-PCR showed that the expression of Seq119 in RSV-inoculated leaves is upregulated slightly at one week post inoculation [23]. Here, to avoid the effect of SBPH during inoculation, we detected the expression of Seq119 in rice leaves systemically infected with the RSV at 40 days post inoculation (dpi). Results showed that its expression level in RSV-infected rice was only 47.1% of that in the uninfected control (Figure 1b). These results show that the expression of Seq119 was downregulated by RSV infection in the RSV systemically infected leaves.

### 2.2. Overexpression of Seq119 in Rice Has No Obvious Effect on Rice Development

**Figure 2:**
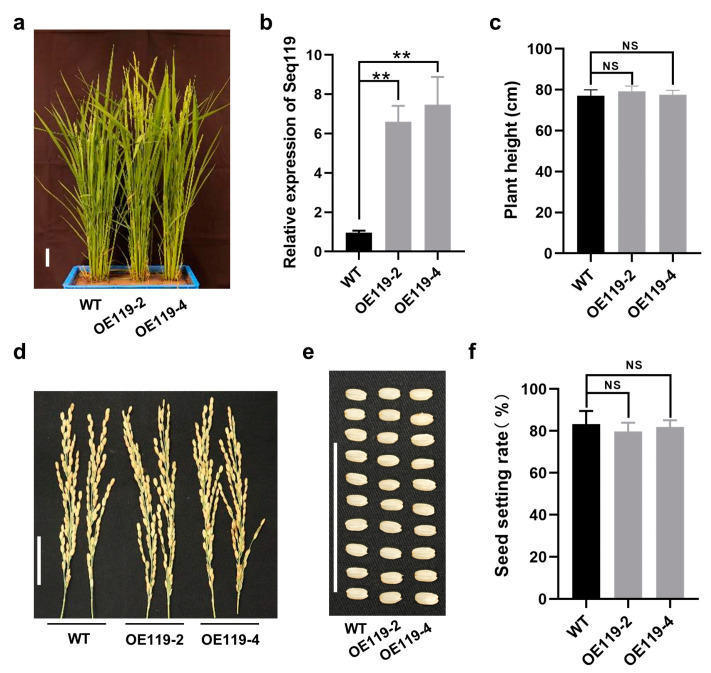
Overexpression of Seq119 in rice plants: (**a**) Two lines of rice plants overexpressing Seq119 (OE119-2 and OE119-4) did not show obvious changes in phenotype. (**b**) Results of qRT-PCR showing the relative expression level of Seq119 in OE119-2 and OE119-4 (**, *p* < 0.01). (**c**) Heights of OE119-2 and OE119-4 plants. (**d**,**e**) Panicles (**d**) and seeds (**e**) of WT plants and OE119-2 and OE119-4 plants. (**f**) Seed setting rate of WT plants and OE119-2 and OE119-4 plants. Bars, 4 cm. NS, no significance.

As a potential novel miRNA, the function of Seq119 in rice plants has not previously been determined. To investigate the possible function of Seq119 in rice, we overexpressed it in rice through the published artificial miRNA method based on the Osa-MIR528 precursor [26,27]. Briefly, the mature sequence of miR528 was replaced with Seq119, and correspondingly, miR528* was replaced with Seq119* in the precursor of miR528. The combined precursor was driven by the 35S promoter and expressed in rice plants through genetic transformation. Two transgenic lines (OE119-2 and OE119-4) were identified with increased expression of Seq119. The expression of Seq119 in the two lines was up to 6–7 times that in control plants (Figure 2a,b). Transgenic plants were not significantly higher than the wild type (Figure 2a,c). In the reproductive phase, transgenic plants showed normal heading, flowering and seeding. Finally, there was no obvious difference in panicle development and seed setting rates between transgenic and wild-type plants (Figure 2c–f). These suggest that the overexpression of Seq119 had no obvious effect on rice development.

### 2.3. Loss of Function of Seq119 Reduces Seed Setting Rate in Rice

**Figure 3:**
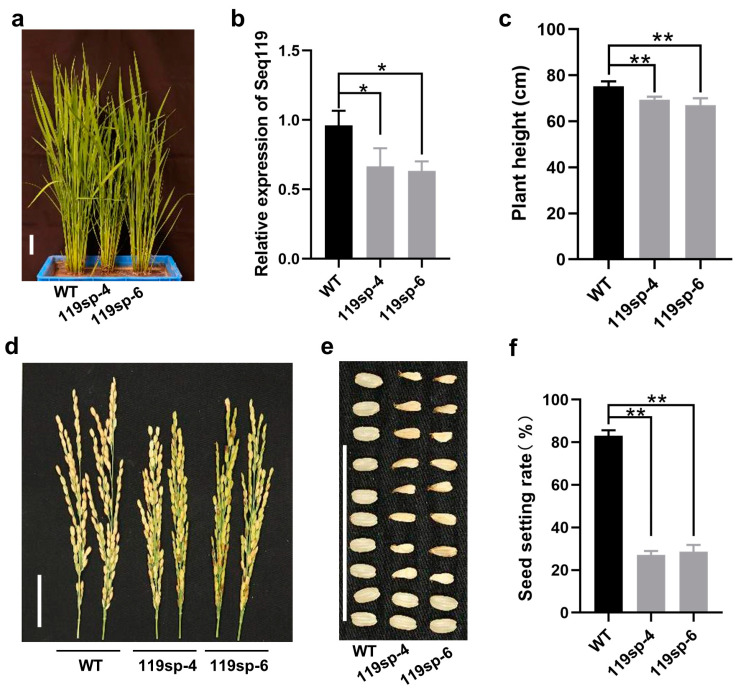
Suppression of Seq119 in rice plants: (**a**) Two lines of plants expressing the target mimic of Seq119 (119sp-4, 119sp-6). (**b**) Results of qRT-PCR showing the relative expression levels of Seq119 in plants (*, *p* < 0.05). (**c**) Heights of 119sp-4 and 119sp-6 plants (**, *p* < 0.01). (**d**,**e**) Panicles (**d**) and seeds (**e**) of WT plants and 119sp-4 and 119sp-6 plants. (**f**) Seed setting rates of WT plants and 119sp-4 and 119sp-6 plants (**, *p* < 0.01). Bars, 4 cm. Values are means ± SD. Student’s *t* test was used to test the significance of the difference.

To further investigate the function of Seq119, we suppressed its function using the improved target mimic method reported before in rice [28,29,30]. The target mimic is a sequence that is designed to bind a specific miRNA via complementation but which cannot itself be cleaved by the miRNA, hence blocking miRNA function [30]. An improved target mimic technique with ability to degrade the miRNAs was also developed [29]. Here, we used the improved target mimic method for analysis. In transgenic plants expressing the target mimic, Seq119 accumulated at a lower level than in wild-type plants, indicating that the target mimic suppressed the function of Seq119 (Figure 3b). Function-suppressed Seq119 plants (119sp-4, 119sp-6) were shorter than the wild type, and the heading was slightly delayed by 3 days (Figure 3a,c). They flowered normally, but only 27.8% and 28.6% of seeds were normal. Moreover, the remainders were not fully developed, resulting in a low rate of seed setting (Figure 3d–f). The results suggest that Seq119 may possibly play a role in seed setting.

**Figure 4:**
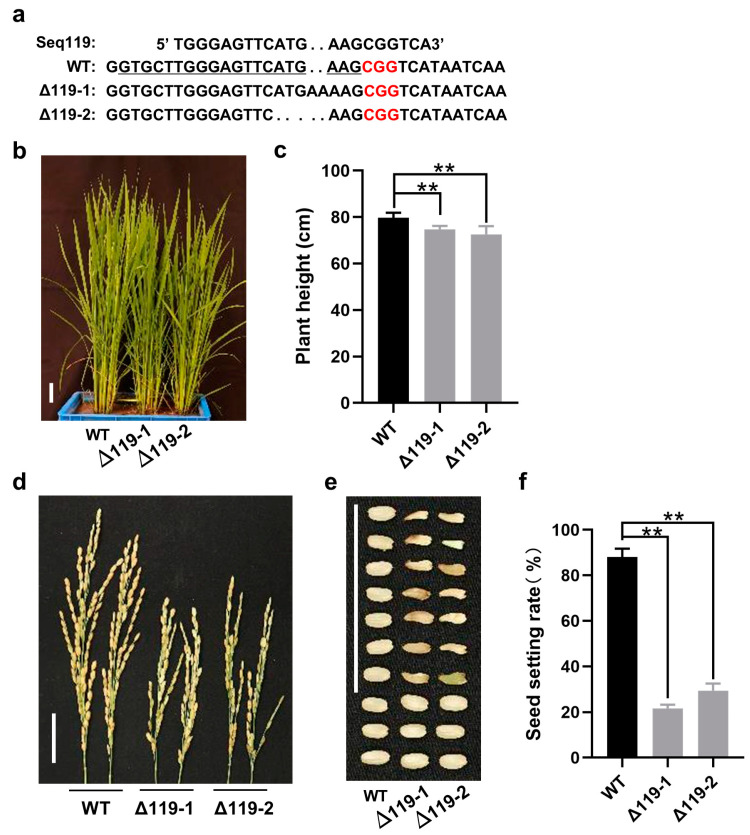
Editing of Seq119 in rice plants: (**a**) Sequence alignment of chromosomal fragment deletion with wild type. (gRNA paired region is labeled with underscored letters, and the PAM region is shown in red letters. The number at the end indicates deleted (−) or inserted (+) bases.) (**b**) Two lines of Seq119-edited plants (Δ119-1, Δ119-2). Bars, 4 cm. (**c**) Plant height of Δ119-1 and Δ119-2 plants (** *p* < 0.01). (**d**,**e**) Panicles (**d**) and seeds (**e**) of WT plants and Δ119-1 and Δ119-2 plants. (**f**) Seed setting rates of WT, Δ119-1 and Δ119-2 plants (**, *p* < 0.01). Bars, 4 cm. Values are means ± SD. Student’s *t* test was used to test the significance of the difference.

To further confirm the function of Seq119, we used the CRISPR-Cas9 system to edit the genomic DNA of Seq119 to knock out its expression. By expressing sgRNA targeting the genomic DNA of Seq119, we finally obtained edited rice homozygous mutants and verified them via Sanger sequencing [31] (Figure 4a). Consistent with 119sp plants, Seq119-edited lines (Δ119-1, Δ119-2) were shorter than the wild type and also had slightly delayed heading in the field (Figure 4b,c). They also flowered normally, but only 21.7 and 29.3% of seeds were normal. Moreover, the remainders were not fully developed, which caused a low seed setting rate (Figure 4d–f).

Thus, the suppression of Seq119 in rice slightly delayed heading and significantly affected seed development, indicating the possible function of Seq119 in seed development.

### 2.4. Identification of the Target Genes of Seq119

**Figure 5:**
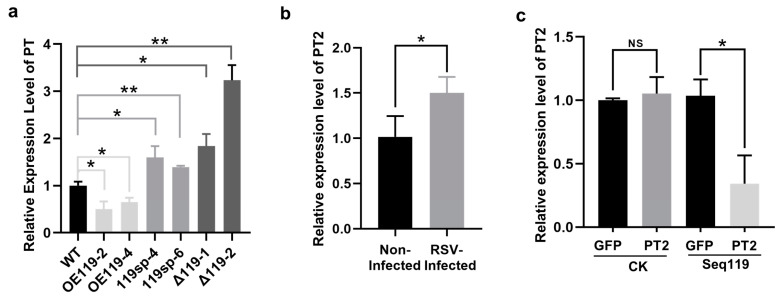
Identification of the target of Seq119: (**a**) Relative expression levels of the predicted target (PT) of Seq119 in WT, OE119, 119sp and Δ119 plants (* *p* < 0.05, ** *p* < 0.01). (**b**) Relative expression levels of *PT2* of Seq119 in RSV-infected rice (* *p* < 0.05). (**c**) Relative expression level of PT2-GFP or mRT2-GFP in analysis expressing Seq119 or a control small RNA (CK) as detected by qRT-PCR (* *p* < 0.05; NS: no significance).

Next, we investigated the potential targets of Seq119. Putative targets for Seq119 were predicted with psRNATarget (https://www.zhaolab.org/psRNATarget/analysis?function=1, accessed on 30 December 2020), using the *Oryza sativa* (rice), transcript, TIGR genome cDNA OSA1 Release 5 (OSA1R5), version 5 as a reference set [32]. Four putative targets were predicted with an expectation smaller than 3.0 (Table 1). We named them as putative target 1-4 (PT1-4). To confirm these targets, we first examined the expression levels of the predicted targets in RSV-infected plants, OE119 plants and 119sp or Δ119 plants. The expression of *PT2* (Accession No: LOC4348735) was upregulated in RSV-infected rice and 119sp or Δ119 plants but was downregulated in plants overexpressing Seq119, which was correspondingly consistent with the expression change in Seq119 in plants (Figure 5a,b). Moreover, the expression of other targets was undetectable or not consistent with the expression change in Seq119 in RSV-infected plants, OE119 plants and 119sp plants, correspondingly (Supplementary Figure S2). Hence, we used *PT2* as the target of Seq119 for analysis.

To confirm the targeting of Seq119 onto *PT2*, we fused green fluorescence protein (GFP) to the C-terminus of *PT2* (PT2-GFP) for analysis. As a control, the mutant *PT2* (mPT2) with mismatch at the binding site of Seq119 was produced and fused with GFP (mPT2-GFP) (Supplementary Figure S3a). Both constructs were cloned behind the ubiquitin promoter of 1300UR to create 1300UR:PT2-GFP and 1300UR:mPT2-GFP. PT2-GFP or mPT2-GFP was co-expressed with Seq119 in leaves of *N. benthamiana* via agroinfiltration. At 3 days post infiltration (dpi), green fluorescence intensity in zones co-expressing PT2-GFP and Seq119 was weaker than that in zones co-expressing mPT2-GFP and Seq119 (Supplementary Figure S3b). Consistently, the transcripts of PT2-GFP in zones co-expressing PT2-GFP and Seq119 accumulated at a lower level than those in zones co-expressing mPT2-GFP and Seq119 (Figure 5d,e). These results demonstrate that Seq119 targets *PT2* for regulation.

### 2.5. Overexpression of PT2 Causes a Low Seed Setting Rate in Rice

**Figure 6:**
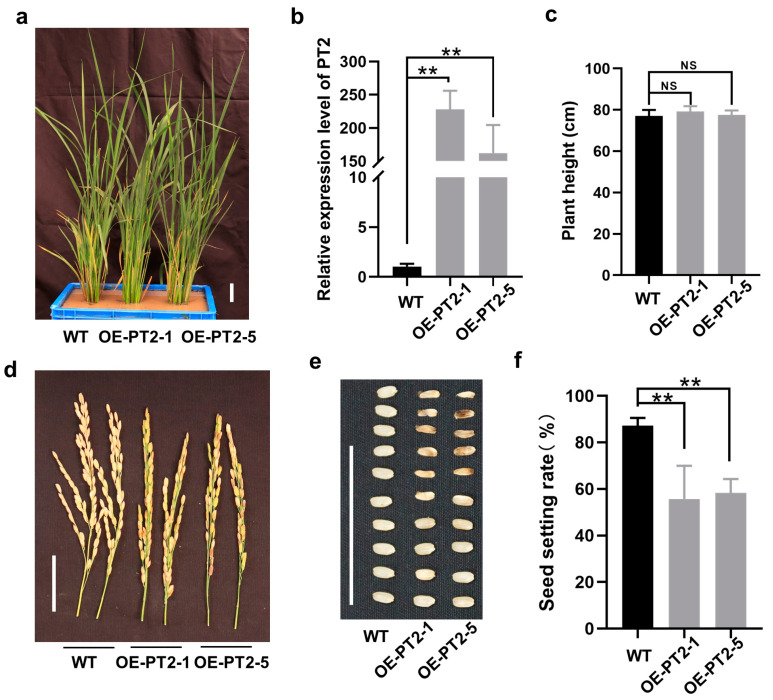
Overexpression of *PT2* in rice: (**a**) Two lines of plants expressing the *PT2* gene (OE-PT2-1 and OE-PT2-5). (**b**) Results of qRT-PCR showing the relative expression levels of *PT2* in plants (** *p* < 0.01). (**c**) Plant height of OE-PT2-1 and OE-PT2-5 plants (NS: no significance). (**d**,**e**) Panicles (**d**) and seeds (**e**) of WT plants and OE-PT2-1 and OE-PT2-5 plants. (**f**) Seed setting rate of WT plants and OE-PT2-1 and OE-PT2-5 plants (** *p* < 0.01). Bars, 4 cm. Values are means ± SD. Student’s *t* test was used to test the significance of the difference.

*PT2* encodes an uncharacterized protein. To determine the biological function of *PT2*, we overexpressed 35S promoter-driven *PT2* in rice via agrobacterium-mediated transformation (Figure 6a). Two independent lines (OE-PT2-1 and OE-PT2-5) were identified with higher expression levels of *PT2* (Figure 6b). Transgenic plants showed normal heading and flowering, but their seed setting rate was affected significantly, with only 55.8–57.3% of seeds filled normally (Figure 6c–f). These results are similar to those obtained with 119sp or Δ119, indicating that *PT2* was targeted by Seq119, contributing to the low seed setting rate symptom in RSV-infected rice plants.

## 3. Discussion

Seq119 is a putative novel miRNA identified in our previous work [23] but for which the biological function was unknown. Here, we provide evidence that the downregulated expression of Seq119 in RSV-infected rice plants contributed to the low seed setting rate symptom in RSV-infected rice plants. MiRNAs play various roles in the growth and development of plants [33,34]. Many studies have reported that viral infection leads to changes in plant miRNA expression, and these changes are considered to be one of the reasons for the appearance of symptoms in infected plants [23,24,35,36,37,38,39]. In our previous work, we found that osa-miR171b is downregulated in RSV-infected rice plants. The inhibition of osa-miR171b causes stunting with reduced chlorophyll content in leaves similar to virus symptoms [24]. The overexpression of osa-miR171b by an artificial miRNA extends vegetative growth and enhances chlorophyll accumulation in leaves [24]. Several reports on other viruses also indicate the involvement of miRNAs in the development of viral symptoms. In a tolerant cassava (TME3), South African cassava mosaic virus (SACMV) infection downregulates 42% of the conserved miRNA families, with highly significant downregulation of miR167 that targets an auxin responsive factor, which plays a role in auxin signaling and adaptive responses to stress, suggesting the importance of auxin signaling in the recovery of SACMV-induced symptoms in TME3 [36]. In an analysis on potato virus Y (PVY)-infected tobacco, the abundance of 18 out of the 26 tested miRNAs was increased upon infection by the severe strains PVY(NTN) and PVY(N-Wi), both of which caused veinal necrosis, but not by the PVY(Z)-NTN strain, which induced milder vein clearing. Furthermore, two miRNAs, nta-miR6020a-5p and nta-miR6164a/b, which target the TIR-NBS-LRR-type resistant TMV *N* genes involved in signal transduction, might correlate with PVY(NTN) and PVY(N-Wi)-induced veinal necrosis [40]. The present data show that the expression of Seq119, a potential novel miRNA, was downregulated in rice infected by RSV for 40 dpi. When the expression of Seq119 was suppressed, rice seed setting rates were extremely low, similar to the effects of RSV infection, which provides additional evidence to support the conclusion that the altered miRNA profile contributes to viral symptoms.

There is increasing evidence that miRNAs function in plant defense against pathogens [41,42,43,44,45,46]. Changed patterns of miRNA expression may reflect plant defense responses against viral infection [47,48,49,50]. Os-miR528 negatively regulates viral resistance in rice by cleaving L-ascorbate oxidase (AO) messenger RNA, thereby reducing the AO-mediated accumulation of reactive oxygen species [50]. Upon viral infection, miR528 becomes preferentially associated with AGO18, leading to elevated AO activity, higher basal reactive oxygen species accumulation and enhanced antiviral defense [50]. Further analysis showed that the miR528-AO defense module is regulated by the transcription factor SPL9 [51]. Moreover, in our previous work, the overexpression of miR171b conferred the tolerance of rice against RSV [24]. Here, it is not known whether the rice plants overexpressing Seq119 could have tolerance or resistance against RSV. We will investigate this next.

miRNAs in plants regulate gene expression through gene silencing, by inducing the degradation of their target messenger RNA or by inhibiting its translation. Here, we identified a potential target of Seq119, *PT2*. Its expression was downregulated in plants overexpressing Seq119 and upregulated in Seq119-suppressed or edited plants. Moreover, fluorescence analysis confirmed the targeting of Seq119 onto *PT2*. These results indicate that *PT2* was regulated by Seq119 through degradation. Moreover, although the other three predicted targets did not seem to be affected by Seq119 in RSV-infected rice, plants overexpressing Seq119 or those where Seq119 was suppressed or edited, we cannot exclude the possibility that they were regulated by Seq119 at the translational level. Additionally, although the phenotype of plants overexpressing *PT2* was consistent with that of Seq119-suppressed or edited plants, the seed setting rate of plants overexpressing *PT2* was not quite at the low levels of Seq119-suppressed or edited plants. These indicate that there may be other pathways regulated by Seq119. It would be interesting to conduct further research into the function of *PT2* and into other pathways or targets regulated by Seq119.

Several miRNAs have been reported to be associated with rice seed development. OsmiR535 and osmiR529a modulate plant height, panicle architecture and grain size by regulating SPL target genes in rice [52,53]. In rice, miR535 is expressed at a very low level during vegetative growth but greatly accumulates in young panicles, similar to osmiR529 [53]. OsmiR535 overexpression increases grain length but does not affect grain width [53]. OsmiR397b regulates a putative AGO protein, OsAGO17, which positively regulates grain size and grain weight [54]. OsmiR530 negatively regulates grain yield. Blocking OsmiR530 increases grain yield, whereas OsmiR530 overexpression significantly decreases grain size and panicle branching, leading to yield losses [55]. Further analysis showed that osmiR530 acts downstream of OsPIL15 [55]. The miR1432-OsACOT (Acyl-CoA thioesterase) module determines grain yield by enhancing the grain filling rate [56]. The suppression of rice miR1432 significantly improved grain weight by enhancing the grain filling rate and led to an increase in the overall grain yield of up to 17.14% in a field trial, indicating huge application potential [56]. Our results show that Seq119, as a putative novel miRNA, is involved in seed development. It is worth analyzing the potential of Seq119 for practical applications next. In the present study, the expression profiles of mRNAs and miRNAs were not analyzed in plants where Seq119 was overexpressed or suppressed. We do not know if the pathway regulating seed development mediated by Seq119 crosses with the previously reported ones, but we now aim to determine the detailed mechanism by which Seq119 functions to regulate seed development.

Taken together, we here demonstrate that the downregulated expression of a small RNA, Seq119, a putative novel miRNA, is associated with a low seed setting rate, a viral symptom of RSV-infected rice. When the expression of Seq119 was suppressed in rice plants either by expressing a mimic target or by CRISPR/Cas editing, seed setting rates were extremely low, similar to the effects of RSV infection. Moreover, a putative target of Seq119 was predicted. The overexpression of the target in rice caused a low seed setting rate, similar to that in Seq119-suppressed or edited rice plants. These results provide additional evidence to support the conclusion that the altered miRNA profile contributes to viral symptoms.

## 4. Materials and Methods

### 4.1. Rice Transformation

The target mimic (TM) technique has proved to be a useful tool for loss-of-function analysis of miRNAs [28,30]. The TM is a sequence that is designed to bind a specific miRNA via complementation but which cannot itself be cleaved by the miRNA, hence blocking miRNA function [30]. An improved TM technique with the ability to degrade miRNAs via a small RNA-degrading nuclease (SDN)-dependent pathway was also developed [29]. Here, the published TM method was used for inhibiting Seq119 activity with minor improvement [29]. Briefly, a TM was designed to contain ten sites for binding Seq119 and was synthesized by Sangon Biotech (Shanghai, China) (Supplementary File S1). At the binding site, the sequence is complementary to Seq119, but ATCT is inserted between the 12th and 13th nucleotides so that the TM binds Seq119 but cannot be cleaved by it. The synthesized TM sequence (MIM119) was cloned into the multicloning sites of binary vector p1300UR to construct the vector 1300UR:MIMSeq119.

The CRISPR-Cas9 system was used in this study as previously described [57,58]. The targeting sequences (Supplementary Table S1) were cloned into the psgR-Cas9-Os vector and were introduced into rice via agrobacterium-EHA105-mediated transformation.

Seq119 was expressed in rice using an osa-MIR528 precursor-based artificial miRNA (amiRNA) [26,27]. This excluded the potential effect of sequences from other parts of the Seq119 precursor. To construct the amiRNA, the osa-miR528 sequence was replaced in the precursor with Seq119 using two primers and was then cloned into the multicloning sites of p1300UR to construct the vector 1300UR:amiR119 (Supplementary File S1).

The constructs were verified via sequencing before being introduced into the Agrobacterium tumefaciens strain EHA105 for transformation. Rice embryonic calli were transformed as previously described [24]. The transgenic seedlings were then transplanted into soil and grown in the greenhouse at 28 °C, 60% relative humidity and a light/dark period of 10 h/14 h. The clay–loam soil was transferred from the field for use.

### 4.2. Plant Materials and Growth Conditions

Plants of rice (*Oryza sativa* L. spp. *japonica*, var Nipponbare) and *Nicotiana benthamiana*, collected in our lab, were used in this study. For the traits assay, the control and transgenic rice plants were grown outdoors in paddy fields with clay–loam soil in Ningbo, China, during the normal rice-growing season from July to October. Plots were 300 square meters (30 m × 10 m) with 20 cm between the plants in the row and 20 cm between rows. *N. benthamiana* plants were grown in a greenhouse at 25 °C, 60% relative humidity and a light/dark period of 14 h/10 h.

### 4.3. Virus Inoculation Assay

RSV-infected rice was prepared as described [25]. Briefly, viruliferous adult brown planthoppers (SBPH) were transferred onto healthy rice seedlings at the three leaf stage for virus inoculation (3 to 5 viruliferous insects per seedling). Control seedlings were inoculated with non-viruliferous planthoppers. After 72 h, the planthoppers were removed. The inoculated plants were grown outdoors in paddy fields with clay soil during the normal rice-growing season from June to October. Plants infected with RSV were tested at 40 dpi via qRT-PCR.

### 4.4. Expression in N. benthamiana Leaves

The vectors, 1300UR:PT-GFP, 1300UR:mPT-GFP and 1301:Seq119, were transformed into Agrobacterium GV3101 via electroporation, grown at 28 °C for 18 h, collected via centrifugation, resuspended in infiltration buffer [10 mM MgCl_2_, 10 mM 2-(N-morpholino) ethanesulfonic acid (MES) and 200 mM acetosyringone, pH 5.6] and kept at room temperature for 3 h. Fully expanded true leaves of *N. benthamiana* were infiltrated with A. tumefaciens resuspension solution (OD600 = 1.0) and were harvested at 72 h post infiltration (hpi) for further research.

### 4.5. Total RNA Extraction and RNA Analysis

Total RNAs were extracted using TRIzol reagent (Invitrogen, USA) according to the manufacturer’s instructions. DNA contamination was removed, and cDNA was synthesized using PrimeScript™ RTreagentKit with gDNA Eraser (Takara, Shiga, Japan). The quality of RNA was checked on a 1% denaturing agarose gel prepared in DEPC-treated water. The rice Actin (*OsActin*) and *N. benthamiana UBC* genes were used as internal references for data normalization. To measure the accumulation of miRNAs, total RNA was reverse-transcribed with the Script miRNA 1st strand cDNA synthesis kit (Tailing Reaction) (SparkJade, Harbin, China) with gDNA Eraser (Takara, Shiga, Japan), and the RT product was subsequently used as a template for quantitative RT-PCR (qRT-PCR) by using miRNA-specific forward primers and the universal reverse primer (Supplementary Table S1). The small nuclear RNA gene *U6* of rice (OsU6) was used as an internal control for the detection of miRNAs. The 2^−∆∆CT^ method was exploited to analyze the relative expression levels of RNAs. All qRT-PCR assays were performed at least three times.

### 4.6. Fluorescence Analysis

The mutated *PT2* was cloned into the binary vector 1300UR at the *Bam*HI and *Kpn*I sites and was introduced into *N. benthamiana* via agrobacterium-strain-GV3101-mediated transformation. Fluorescence signals were monitored using a Nikon A1+ confocal laser scanning microscope system (Tokyo, Japan). At least three independent biological repeats were conducted with similar results.

## Figures and Tables

**Table 1:** Predicted targets of Seq119.

Targets	Target Acc.	Expectation	Target Description
*PT1*	LOC4352810	1	uncharacterized protein
*PT2*	LOC4348735	2.5	uncharacterized protein
*PT3*	LOC4348324	3	uncharacterized protein
*PT4*	LOC4325059	3	TRANSPARENT TESTA 1

## Data Availability

The data are contained within the article or Supplementary Material.

## Supplementary Materials

The supporting information can be downloaded at https://www.mdpi.com/article/10.3390/ijms24043675/s1.
