# Supplementary material for: A Novel miRNA in Rice Associated with the Low Seed Setting Rate Symptom of Rice Stripe Virus
Source: Int J Mol Sci. 2023 Feb 12;24(4):3675. doi: 10.3390/ijms24043675 (PMC9967548; doi:10.3390/ijms24043675)
Supplement: Supplementary file 1 [file ijms-24-03675-s001.zip › File S1 The sequences of target mimic and overexpressing Seq119.pdf]

The sequences of target mimic

GAGCTC**TGACCGCTTCAATCTGAACTCCCA**AGCT**TGACCGCTTCAATCTG**
**AACTCCCA**AGCT**TGACCGCTTCAATCTGAACTCCCA**AGCT**TGACCGCTTC**
**AATCTGAACTCCCA**AGCT**TGACCGCTTCAATCTGAACTCCCA**AGCT**TGAC**
**CGCTTCAATCTGAACTCCCA**AGCT**TGACCGCTTCAATCTGAACTCCCA**AG
CT**TGACCGCTTCAATCTGAACTCCCA**AGCT**TGACCGCTTCAATCTGAACT**
**CCCA**AGCT**TGACCGCTTCAATCTGAACTCCCA**GGATCC

The sequences of artificial Seq119 (overexpressing Seq119)

GAGCTCTTTGGCTGTAGCAGCAGCAG**TGGGAGTTCATGAAGCGGTCA**CAGG
AGATTCAGTTTGAAGCTGGACTTCACTTTTGCCTCTCTTGACCGCTTCATGAA
CTCCCATTCCTGCTGCTAGGCTGTTCGGATCC
