# Supplementary material for: A Novel miRNA in Rice Associated with the Low Seed Setting Rate Symptom of Rice Stripe Virus
Source: Int J Mol Sci. 2023 Feb 12;24(4):3675. doi: 10.3390/ijms24043675 (PMC9967548; doi:10.3390/ijms24043675)
Supplement: Supplementary file 1 [file ijms-24-03675-s001.zip › Suppl. Figures.pdf]

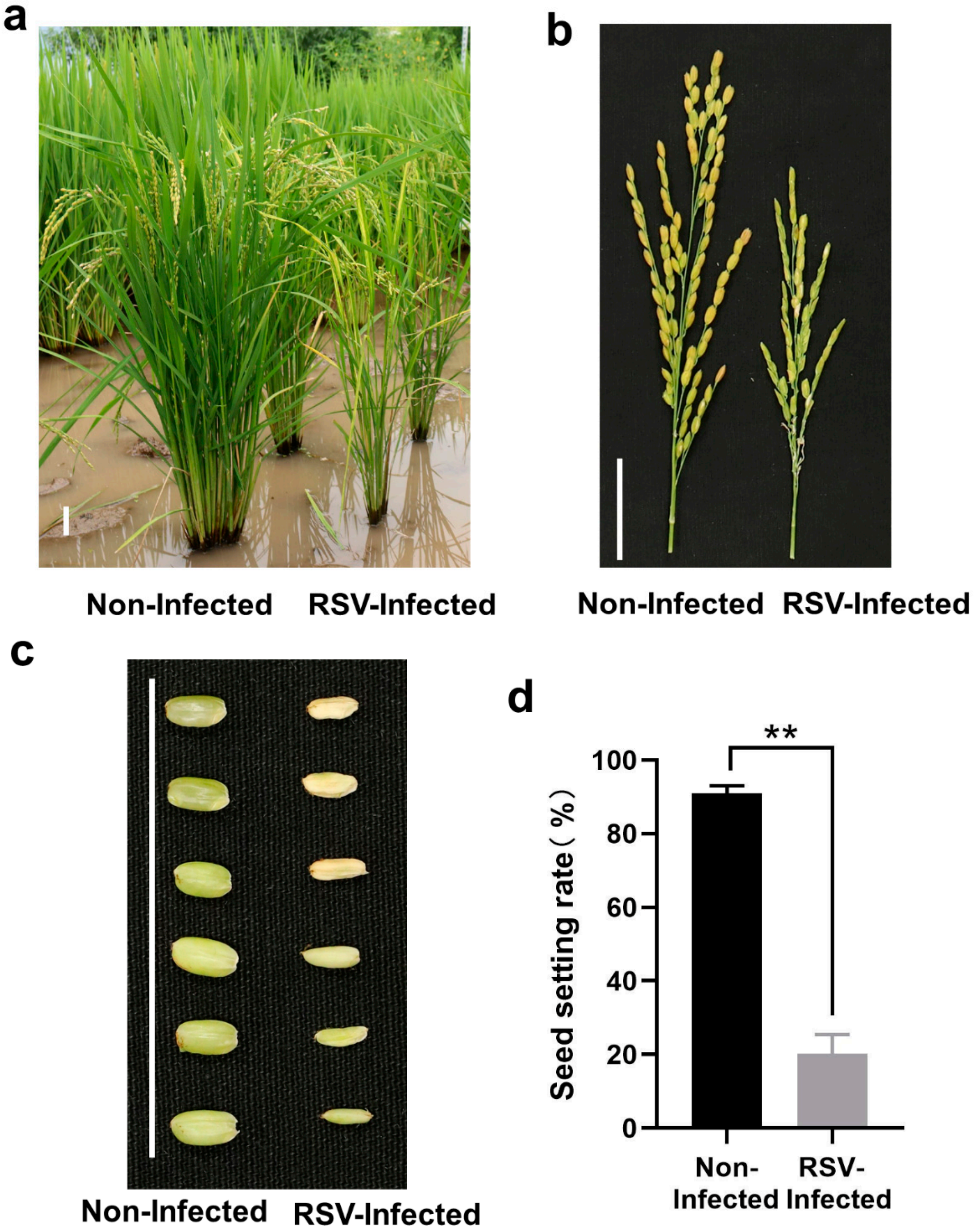

Supplementary Figure S1 RSV infection caused the low seed setting rate of rice. (a) Plants infected by RSV in the field. (**b and c**) Panicle (b) and seeds (c) of rice infected by RSV. **(d)** Seed setting rate of RSV-infected rice decreased significantly (**, p<0.01). Values are means ± SD. Student's *t* test was used to test the significance of the difference. Bars, 4 cm.

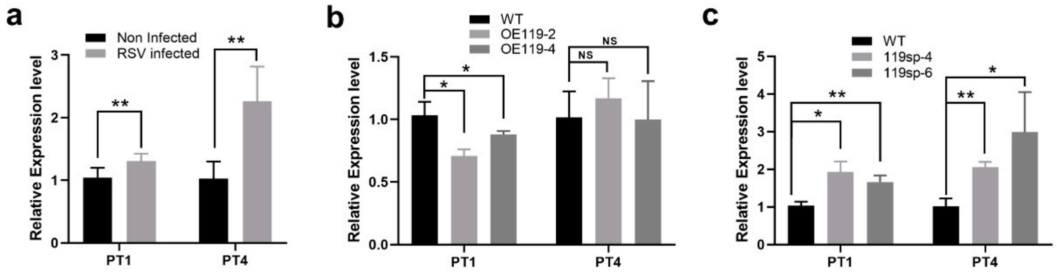

Supplementary Figure S2: Expression of targets of Seq119 in in RSV-infected plants (a), OE119 plants (b) and 119sp plants (c). Expression of *PT3* was undetectable in leaf samples, hence not shown in results. Values are means ± SD. Student's *t* test was used to test the significance of the difference (*, p<0.05; **, p<0.01; NS, No significance).

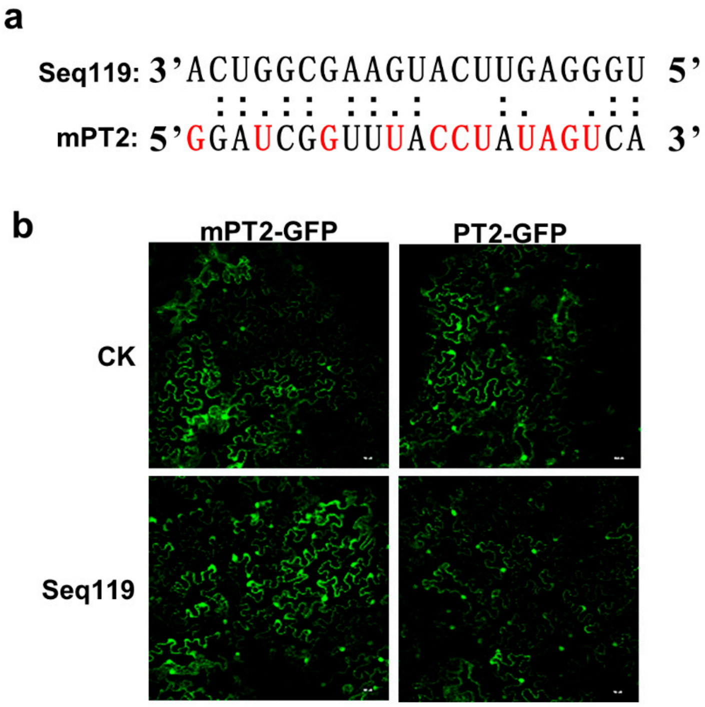

Supplementary Figure S3 Analysis of the target of Seq119. (a) the binding site was mutated on the PT2, producing mPT2 for analysis. The red letters indicate the changed nucleotides at the binding site of Seq119. (**b**) PT2-GFP or mPT2-GFP

were co-expressed with Seq119 for analysis in epidermal cells of *N. benthamiana*. Confocal images were taken at 60 h after Agrobacterium infiltration. miR528 was used as control (CK).
